# A gamification-based intervention to encourage active travel

**DOI:** 10.1093/eurpub/ckac129.586

**Published:** 2022-10-25

**Authors:** M Harris

**Affiliations:** Intelligent Health, Cardiff, UK

## Abstract

**Purpose:**

‘Beat the Street’ is a community-wide intervention which aims to increase active travel by turning an area into a 6-week game. Residents earn points and prizes by walking and cycling and tapping a smartcard on RFID readers called ‘Beat Boxes’ placed on lampposts at half-mile intervals. To-date, over 1 million people have taken part in the intervention, however, the impact of the program on adult active travel is yet to be explored.

**Methods:**

In Autumn 2019, Beat the Street was delivered throughout the London Borough of Hounslow. Prior, and immediately following the intervention, residents were invited to complete a self-report questionnaire (Sport England Active Lives Survey-SF) to assess changes in physical activity. Time-stamp data generated through Beat Box activity provided an objective measure of intervention engagement and a traffic survey camera was used to measure the number of cars travelling along 1 target road between 1-week pre- and 1-week post-intervention.

**Results:**

28,219 people took part in the six-week game. Between pre- and post-intervention there was 7% decrease in adults reporting less than 30mins of activity per week and a 13% rise in adults reporting 150+ mins (n = 346, p < 0.01). Beat box data ascertained that 25% of total taps at all Beat Boxes were made between 08:00-08:59am and a further 28% were made between 3:00-3:59pm, typical travel to school/work periods. Further, traffic camera data showed that between the week before and week following Beat the Street, 1199 and 705 fewer cars and 130 and 36 fewer vans were observed travelling along Cambridge Road between 07:00-09:30am and 2:00-4:30pm, respectively.

**Conclusions:**

These data sources, in combination, suggest gamification may be an encouraging approach to increasing levels of active travel at a community-wide level.

**Key messages:**

• Gamification based intervention increases physical activity.

• Gamification encourages active travel to school and work.

